# Plant-fed versus chemicals-fed rhizobacteria of Lucerne: Plant-only teabags culture media not only increase culturability of rhizobacteria but also recover a previously uncultured *Lysobacter* sp., *Novosphingobium* sp. and *Pedobacter* sp.

**DOI:** 10.1371/journal.pone.0180424

**Published:** 2017-07-07

**Authors:** Nabil A. Hegazi, Mohamed S. Sarhan, Mohamed Fayez, Sascha Patz, Brian R. Murphy, Silke Ruppel

**Affiliations:** 1 Environmental Studies and Research Unit (ESRU), Department of Microbiology, Faculty of Agriculture, Cairo University, Giza, Egypt; 2 Leibniz Institute of Vegetable and Ornamental Crops Großbeeren/ Erfurt e.V. (IGZ), Großbeeren, Germany; 3 Department of Botany, School of Natural Sciences, Trinity College Dublin, Dublin, Ireland; University of the West of England, UNITED KINGDOM

## Abstract

In an effort to axenically culture the previously uncultivable populations of the rhizobacteria of Lucerne (*Medicago sativa* L.), we propose plant-only teabags culture media to mimic the nutritional matrix available in the rhizosphere. Here, we show that culture media prepared from Lucerne powder teabags substantially increased the cultivability of Lucerne rhizobacteria compared with a standard nutrient agar, where we found that the cultivable populations significantly increased by up to 60% of the total bacterial numbers as estimated by Quantitative Real-time Polymerase Chain Reaction (qRT-PCR). Cluster analysis of 16S rDNA Polymerase Chain Reaction-Denaturing Gradient Gel Electrophoresis (PCR-DGGE) of cultivable Colony-Forming Units (CFUs) revealed a more distinct composition and separation of bacterial populations recovered on the plant-only teabags culture media than those developed on a standard nutrient agar. Further, the new plant medium gave preference to the micro-symbiont *Sinorhizobium meliloti*, and succeeded in isolating a number of not-yet-cultured bacteria, most closely matched to *Novosphingobium* sp., *Lysobacter* sp. and *Pedobacter* sp. The present study may encourage other researchers to consider moving from the well-established standard culture media to the challenging new plant-only culture media. Such a move may reveal previously hidden members of rhizobacteria, and help to further explore their potential environmental impacts.

## Introduction

It has been long established that more than 90% of environmental microorganisms are not amenable to cultivation on/in standard laboratory media [1]. Bacterial databases are continually updated with new bacterial taxa, most of which are not yet cultured. Microbial ecologists struggle to recover and maintain these unculturables as viable entities grown as pure cultures. The last decade has witnessed the development of culture media and conditions for culturing new bacterial members from various environments [2–8]. In 2012, we proposed the use of plant-only culture media as a new approach for culturing rhizobacteria that might resist conventional cultivation. Crude plant juices, saps and powders, without any amendments, supported good growth and recoverability of rhizobacteria, comparable with the standard culture media [9–11]. For ease of application and practicability, tea bags packed with plant powders were successfully introduced to obtain the plant infusions necessary to prepare culture media [11].

The host plant tested was Lucerne (alfalfa, *Medicago sativa* L.), being the well-established crop available in the experimental open fields of IGZ-Großbeeren. Among other perennial and/or annual legume crops, it is known to be the most common grown forage because of its ability to be grown over a range of climatic conditions, efficient yield, nutritional qualities and biotic (e.g. pests) and abiotic stress-tolerance (e.g. salinity and soil acidity) [12]. Globally, Lucerne is used as a major source of protein for livestock and in crop rotation as pasture and organic/green manure fertilization. It is of very significant contribution to the N-status and biofertility of the plant-soil system via the consortium of rhizobacteria, namely the nodule-forming rhizobia and associated plant growth-promoting bacteria.

As to the molecular aspects of macro- and micro-symbiosis, *Medicago sativa* and its relative *Medicago truncatula* (Gaertn.) are commonly used as model legume plants. Very much emphasis was particularly devoted to the level of Alphaproteobacteria species *Sinorhizobium meliloti* [13], not to the whole bacterial community composition. Later, Pini *et al*. [14] used culture-independent techniques (T-RFLP, qPCR and 16S rRNA sequencing), and showed a high diversity; more than 7 classes with the distinguished dominance by members of Alphaproteobacteria in the plant-soil system including soil and plant shoots. Among the Alphaproteobacteria families, Sphingomonodaceae and Methylobacteriaceae were abundant inside plant tissues, including aerial parts, while in soil they were represented by Hyphomicrobiaceae, Methylocystaceae, Bradirhizobiaceae and Caulobacteraceae. On the species level, *S*. *meliloti* were detected with higher values in root nodules compared to those reported in soil and aerial tissues of leaves and stems. Further, the species is proved to behave as an endophytic strain colonizing all plant compartments, besides being a root symbiont of legumes [15]. Also, PCR-based 454 pyrosequencing confirmed the decreasing bacterial diversity from unplanted soil to root tissues of Lucerne, and that the root tissues were mainly inhabited by Alphaproteobacteria associated by Gammaproteobacteria, as well as Betaproteobacteria, Flavobacteria and Actinobacteria, which may have some additional plant-growth-promoting activities [14,16]. Of interest was the study of Schwieger and Tebbe [17] who compared the products of cultivated isolates and community profiles as determined by Cultivation-independent-SSCP (Single Strand Conformation Polymorphism) and indicated that the most dominant groups identified by cultivation were also detected in the community profiles. However, there was one exception where the most dominant group, which represented 24% of the total cultivated isolates and was related to the genus *Variovorax*, was not detected. These results together with those of Dunbar *et al*. [18] raise questions and reservations towards the inherent problems and limitations of the existing culture-independent techniques. And, strongly encourage the on-going sincere efforts to improve culturability of the plant microbiome.

In the present study, we tested the suitability of culture media prepared from teabags packed with varying quantities of dehydrated powder of Lucerne (*Medicago sativa* L.) for culturing host rhizobacteria. Quantitative real-time PCR (qPCR) was applied to measure the total bacterial copy numbers present on Lucerne roots to assess the relative efficiencies of cultivability on tested culture media. A replica plating technique was used to test reproducibility of developed CFUs on various culture media. PCR-DGGE analysis of cultured bacterial populations and 16S rDNA sequencing were carried out to identify and compare the cultivable rhizobacterial community composition.

## Material and methods

### Host plant and sampling

Intact shoots and roots of Lucerne plants (*Medicago sativa* L.) were randomly sampled from the experimental fields of Leibniz institute of vegetable and ornamental crops (IGZ, 52°20'56.2"N—13°19'00.6"E), Großbeeren, Germany. Roots were initially washed with tap water and aliquoted for bacterial CFU (fresh root material) and qPCR measurements (freeze dried). Root dry weight was determined (drying at 65°C for 24 hrs.) to calculate the bacterial counts per gram dry root. The plant vegetative parts were kept for the preparation of the plant-only teabags culture media.

### Culture-independent quantification of Lucerne rhizobacteria

#### Total bacterial quantification using quantitative real-time PCR

Total DNA (bacterial and plant DNA) was extracted from freeze dried Lucerne roots, using QIAGEN DNeasy plant mini kit (QIAGEN, Hilden, Germany) according to the manufacturers’ instructions. The quality of DNA isolated from roots using DNeasy Plant MiniKit (Qiagen) was determined photometrically by the 260/280 ratio calculation to be above 1.9, and the A_230_ measurement was nearly 0 and quantified at 260 nm. Detection and quantification of bacterial 16S rDNA copy numbers were performed by quantitative real-time PCR (qPCR) using the CFX96 Touch^™^ Detection System (Bio-Rad Inc., CA, USA) in optical grade 96 well plates. The PCR reaction was performed according to Sarhan *et al*. [11] in a total volume of 25 μl using SYBR^®^ green master mix (Bio-Rad) containing 10–30 ng genomic DNA and 8.25 pmol of each primer [19]; the universal forward 519f (CAGCMGCCGCGGTAANWC) and reverse 907r (CCGTCAATTCMTTTRAGTT). The cycling program and both standard and melting curves construction were done according to Sarhan *et al*. [11]. Bacterial cell numbers were indirectly calculated assuming an average 16S rDNA copy number of 3.6 per bacterial cell [20,21].

### Culture-dependent quantification and characterization of Lucerne rhizobacteria

#### Culture media preparation

**Fig 1:**
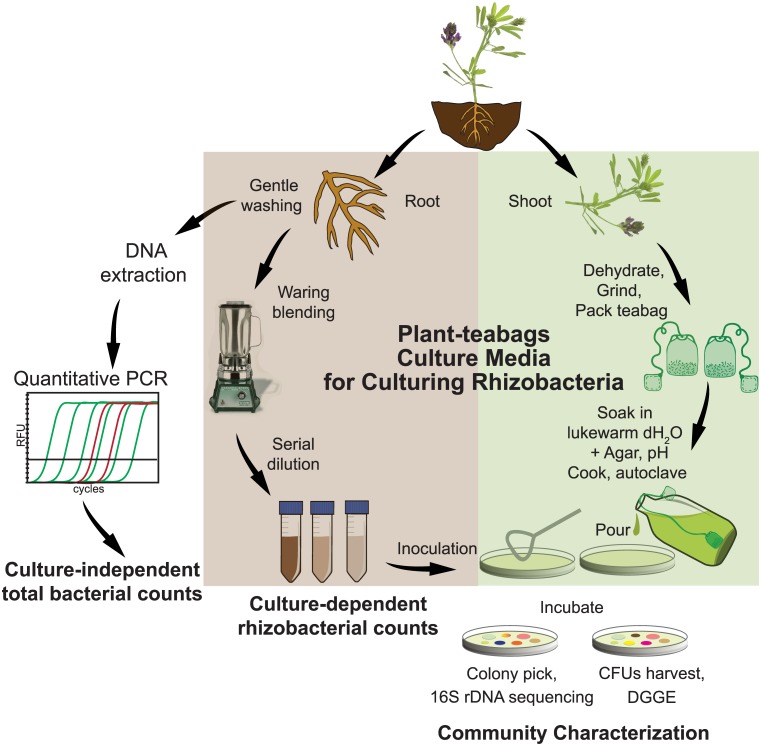
Schematic illustration for the preparation of plant-only teabags culture media, and the work flow of culture-dependent and culture-independent analyses of Lucerne rhizobacteria.

Plant-only teabags were prepared according to Sarhan *et al*. [11]. Fresh vegetative parts, including leaves and stems, of Lucerne were oven dried at 65°C for 18 hrs. Then the dry plant material was mechanically ground and passed through a 2.0 mm sieve to obtain a fine dehydrated powder. Teabags were packed with different quantities of the prepared dehydrated Lucerne powder: 0.25, 0.5, 1.0 and 4.0 g per pouch. The teabags were soaked in lukewarm distilled water to obtain plant infusions of 0.25, 0.5, 1.0 and 4.0 g l^-1^, and pH was neutralized to 7.0. The teabags were left during autoclavation for further extraction. Agar culture media were prepared by adding agar (2%, w/v) and autoclaved at 121°C for 20 min. A schematic illustration of plant-only teabags and culture media preparation is presented in Fig 1.

The nutrient agar Standard-I, with the addition of glucose (1.0 g l^-1^), (Merck KGaA, Darmstadt, Germany) was used as the control.

#### Culture-dependent recovery of *in-situ* rhizobacteria of Lucerne

Plant roots were initially washed with tap water, then with sterilized water before crushing in a previously autoclaved Warring blender, with an adequate volume of autoclaved basal salts of the yeast mannitol agar (YMA) medium [22] as diluent. Further serial dilutions were prepared, and 200 μl aliquots of suitable dilutions were surface inoculated on agar plates, with 3 replicates, prepared from the plant-only teabags (0.25, 0.5, 1.0, 4.0 g l^-1^) and the Standard-I nutrient agar (Merck, Darmstadt, Germany). The culture plates were incubated at 28°C and CFUs were counted throughout the incubation period at two, four and fifteen days. Bacterial counts were log transformed, tested for equality of variances, and a 2-way ANOVA was performed using STATISTICA 10 (Statsoft inc. 2011).

#### Harvest of CFUs and DNA extraction

For DNA extraction, all CFUs developed on representative agar plates (> 30–300 CFU plate^-1^) of the tested culture media were washed using 0.05 M NaCl solution, and collected by centrifugation for 10 min. at 9500 *g*. DNA was extracted from collected CFUs pellets using the QIAGEN DNeasy plant mini kit (QIAGEN, Hilden, Germany) according to the manufacturers’ instructions. DNA concentrations and quality were checked as previously described.

### 16S rDNA PCR-DGGE fingerprinting

Bacterial DNA, extracted from the total CFUs harvested from the agar plates, was used to amplify the whole 16S rDNA gene using the primers 9bfm (GAGTTTGATYHTGGCTCAG) and 1512r (ACGGHTACCTTGTTACGACTT) [23]. The reaction was performed using the Bio-Rad C1000 Thermal Cycler (Bio-Rad, Hercules, CA, USA) in a total volume of 25 μl using QIAGEN TopTaq master mix (Qiagen Inc., Hilden, Germany), containing 10–30 ng genomic DNA and 8.25 pmol of each primer. The PCR thermal cycling program was adjusted according to Sarhan *et al*. [11]. A QIAquick PCR Purification Kit (Qiagen Inc., Hilden, Germany) was used to purify the PCR product, according to the manufacturers’ instructions. For amplification of the V3 region, a nested PCR was performed using 341f-GC (CGCCCGCCGCGCGCGGCGGGCGGGGCGGGGGCACGGGGCCTACGGGAGGCAGCAG) and 518r (ATTACCGCGGCTGCTGG) primers [23,24]; the reaction conditions and thermal cycling program used were as described in Sarhan *et al*. [11]. PCR products of the V3 region were heated at 95°C for 5 min and stored at 65°C before loading onto the gradient gel. The products of both PCR reactions were tested on 1.5% agarose gel to ensure single products of the expected size.

DGGE was performed using the Dcode Mutation Detection System (Bio-Rad Inc., CA, USA). PCR products (10 μ1 of 10–15 ng PCR products mixed with loading dye) were electrophoresed on 8% polyacrylamide gel containing 30 to 70% denaturing gradient of formamide and urea with 1x TAE buffer. DGGE was conducted at 60°C for 20 hrs at a voltage of 50 V. The gel was stained with SYBR^®^ Gold Nucleic Acid Gel Stain (Life Technologies Inc., Germany) and recorded with a UV gel documentation system (Biometra GmbH, Goettingen, Germany). A self-created standard was constructed according to Sarhan *et al*. [11]. The DGGE fingerprints were analyzed using Phoretix 1D pro software (TotalLab Ltd. v.11.4). Principal coordinates analysis (PCoA) was performed using GenALEx 6.5 [25].

#### Replica plating and 16S rDNA sequencing

Plates with confined colonies (≤ 30 plate^-1^), developed with the longer incubation of 15 days, were replicated on agar plates of homologous and heterologous culture media [26]. With incubation at 29°C, reproduced colonies were further subcultured on agar plates of the corresponding culture medium. The two groups of both common colonies and those only confined to plant-only teabags culture media were subjected to 16S rDNA sequencing (Eurofins MWG Operon, Ebersberg, Germany). To obtain the nearest phylogenetic neighbours, 16S rDNA sequences were compared with their closest matches using BLASTn tool (blast.ncbi.nlm.nih.gov/Blast.cgi)[27] and the classifier tool of Ribosomal database project database (rdp.cme.msu.edu/classifier/classifier.jsp)[28], executed on April 05, 2016. The Sequence alignment and the phylogenetic tree were constructed using ClustalW and the Neighbors-Joining method based on the Jukes-Cantor model implemented in MEGA 6.0 [29]. *Bacillus* species were selected as an out-group. The bootstrap values were calculated after 1000 replicates and indicated at each node. The 16S rDNA sequences identified in this study have been deposited in the GenBank database under the accession numbers KP411236, KR911852, KP411237, KR911853, KP411238, KR911855, KR911854, KP411239, KP411240, KP411241, KP339867 and KP411242.

## Results

### Quantification of Lucerne rhizobacteria using culture dependent and independent techniques

**Fig 2:**
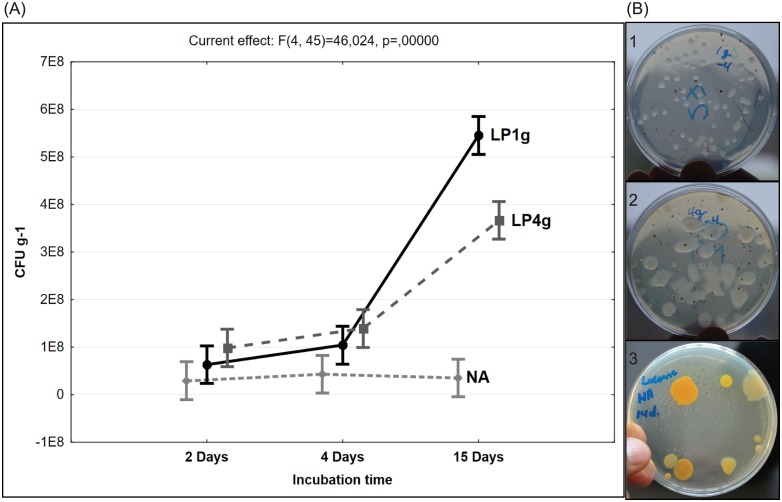
Cultivable rhizobacteria of Lucerne roots (CFUs g^-1^ root) developed on all tested culture media. A, ANOVA analysis (2-way interactions) of CFUs numbers of Lucerne rhizobacteria as affected by culture media and incubation period. NA, nutrient agar; LP1g, teabags of Lucerne powder 1.0 g l^-1^; LP4g, teabags of Lucerne powder 4.0 g l^-1^. B, Morphology of CFUs developed on agar plates of culture media prepared from teabags-only-containing Lucerne powder (4.0 g l^-1^ and 1.0 g l^-1^ culture media) as well as nutrient agar, inoculated with aliquots of 200 μl of the same root suspension (10^−4^): B1, confined/limited and transparent/translucent colonies on LP1.0 g l^-1^; B2, numerous slimy colonies on LP4.0 g l^-1^; B3, large tinted colonies on NA.

Surface-inoculated agar plates were used to estimate the cultivable population (CFUs numbers) of rhizobacteria of Lucerne plants. Agar plates were prepared from both Standard I nutrient agar and plant-only teabags culture media, where teabags contained 4.0, 1.0, 0.5 or 0.25 gram dehydrated Lucerne powder per liter. Compared with the Standard I nutrient agar, all tested plant powder contained greater quantities of nutrients, and supported excellent development of macro- and micro-colonies of rhizobacteria. We found numerous transparent/translucent and slimy bacterial colonies growing on the 4.0 g l^-1^ Lucerne powder medium, while a greater number of smaller colonies developed on the 1.0 g l^-1^ Lucerne powder medium. In contrast, the colonies growing on the Standard I nutrient agar were remarkably tinted and relatively sparse (Fig 2). Numbers of CFUs were significantly different (P < 0.05) between plant-only teabags culture media and Standard I nutrient agar, and increased with incubation time particularly on plant-only teabags culture media (Table 1). Longer incubation (15 days) increased the numbers of micro-colonies, particularly on plant-only teabags culture media and culminated in significant increases in the total number of CFUs. Mean log CFU counts were highest for plant-only teabags containing 1.0 g l^-1^ (8.73 ± 0.018 log CFU g^-1^ root) followed by 0.50, 0.25 and 4.0 g l^-1^ (8.51 ± 0.023 log CFU g^-1^ root), and were lowest for Standard I nutrient agar (7.31 ± 0.055 log CFU g^-1^ root) (Table 1). Total bacterial cell numbers were estimated using qPCR analysis of 16S rDNA copy numbers per gram of dry Lucerne roots. Assuming an average of 3.6 16S rDNA copy numbers per bacterial cell [20,21], the mean total bacterial cell numbers amounted to 8.94 ± 0.041 per gram of Lucerne root. Relating CFU numbers to the total bacterial numbers measured by qPCR revealed higher culturability efficiencies on plant-only teabags culture media (62–71%) compared to the Standard I nutrient agar (2–16%), particularly with the extension of incubation period up to 15 days (Table 1).

**Table 1:** The culturability of Lucerne rhizobacteria on various culture media.

**Experiment 1. Nutrient agar compared to Lucerne teabags of 1.0 and 4.0 g l**^**-1**^ **culture medium**				
Culture media	log CFU count g^-1^ root (at 2 day incubation time) **	Culturability (%)*	log CFU count g^-1^ root (at 15 day incubation time)**	Culturability (%)*
Nutrient agar	7.180 ± 0.055^d^	2%	7.309 ± 0.035^d^	2%
Lucerne powder 4.0 g l^-1^	7.911 ± 0.099^c^	9%	8.511 ± 0.023^b^	37%
Lucerne powder 1.0 g l^-1^	7.548 ± 0.037^cd^	4%	8.737 ± 0.018^a^	62%
**Experiment 2. Nutrient agar compared to Lucerne teabags of decreasing quantities of Lucerne powder, 1.00, 0.50, 0.25 g l**^**-1**^ **culture media**				
Culture Media	Log CFU g-1 root at 15 days of incubation**			Culturability (%)*
Nutrient agar	8.03 ± 0.085^b^			16%
Lucerne powder 1.00 g l^-1^	8.71 ± 0.042^a^			71%
Lucerne powder 0.50 g l^-1^	8.69 ± 0.048^a^			71%
Lucerne powder 0.25 g l^-1^	8.64 ± 0.055^a^			66%

* Culturability calculated as numbers of CFUs developed on agar plates and related to the total bacterial numbers measured by quantitative real-time PCR. The mean value of qPCR cell numbers indirectly obtained for 8 replicates is log 8.93 ± 0.041 g^-1^ root dry weight (2 plant biological samples with 4 technical replicates, and assuming that the average 16S rDNA copy number per bacterial cell is 3.6 [20,21].

** Data are log means ± standard error (SE), n = 3. Statistical significant differences are indicated by different superscript lowercase letters (*P* value ≤ 0.05, n = 3).

### PCR-DGGE fingerprinting of Lucerne culturable rhizobacteria

**Fig 3:**
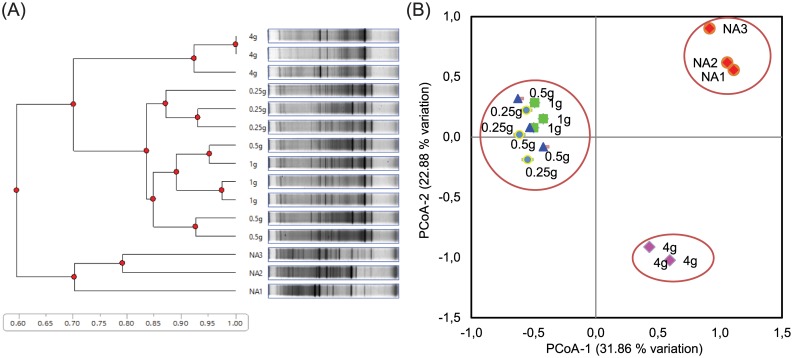
Cluster analysis of culturable Lucerne rhizobacteria (CFUs) developed on all tested culture media. A, UPGMA cluster analysis, with Euclidean distances, of 16S rDNA-DGGE pattern obtained for culturable populations (CFUs) developed on agar plates of culture media prepared from Lucerne powder-only-teabags (4.0 g l^-1^, 1.0 g l^-1^, 0.5 g l^-1^ and 0.25 g l^-1^ Lucerne powder) as well as standard I nutrient agar (NA). B, The principal coordinate analysis of the obtained DGGE fingerprints.

Based on UPGMA cluster analysis of 16S rDNA PCR-DGGE band patterns obtained for the cultivable CFU populations, a distinct separation at a similarity level of 60% was distinguished between Standard I nutrient agar and all Lucerne plant-only teabags culture media. With higher levels of similarities, 70% and >85%, successive separations were attributed to the tested quantities of plant powders in the teabags used for the preparation of culture media (Fig 3A). In addition, the principal coordinate analysis of the obtained DGGE fingerprints revealed three main clusters along the first axis representing 31.86% of variation; one cluster included the lower quantities of Lucerne powder (1.0, 0.50, and 0.25 g l^-1^) and the second cluster for the higher quantity of 4.0 g l^-1^. The third cluster was for the Standard I nutrient agar alone. The second axis representing 22.88% variation showed the same trend of clustering (Fig 3B). This is a strong indication of the independent effects of culture media type and concentrations of nutrients on the culturability and community composition of rhizobacteria.

### Replica plating of rhizobacteria colonies and 16S rDNA sequencing

All of the tested 122 colonies grown on Standard I nutrient agar were successfully reproduced on the plant-only teabags culture medium (1.0 g l^-1^). In general, colonies developed on plant-only teabags culture media were transparent to translucent, and when reproduced on nutrient agar exhibited colored pigmentation. Among 184 of those colonies originally developed on such plant-only teabags medium, some failed to reproduce on Standard I nutrient agar. A total of 12 morphologically different colonies were traced and followed: ten grew only on plant-only teabags culture media and the other two were able to grow on either media.

**Fig 4:**
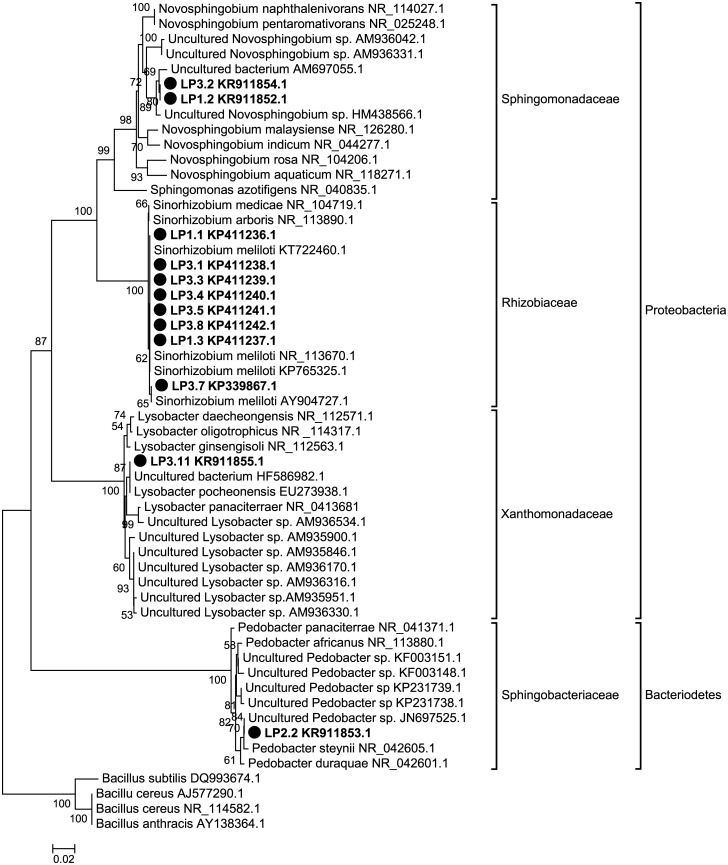
*Medicago sativa* rhizobacteria isolates phylogeny based on 16S rDNA sequences. The isolates of this study are indicated by solid circle (●) (LP1.1, LP1.2, LP1.3, LP2.2, LP3.1, LP3.2, LP3.3, LP3.4, LP3.5, LP3.7, LP3.8, and LP3.11), and closest matches obtained from the GenBank database are indicated by accession numbers after organism name, using Neighbor-Joining method with calculated Jukes-Cantor distances. *Bacillus* spp. were included as an out-group to root the tree. The node numbers return the bootstrap values after 1000 replicates.

16S rDNA sequencing of these pure isolates confirmed that among the ten colonies that only reproduced on plant-only teabags media, eight (LP1.1, LP1.3, LP3.1, LP3.3, LP3.4, LP3.5, LP3.7, and LP3.8) were identified as *Sinorhizobium meliloti*, phyla: Proteobacteria, class: Alphaproteobacteria. The remaining two colonies (LP1.2 and LP3.2) were identified as phyla: Proteobacteria, class: Alphaproteobacteria, and both were matching closely, according to BLASTn tool of the Genbank database, to an uncultured Bacterium (AM697055.1) and/or to an uncultured *Novosphingobium* sp. (HM438566.1) (Fig 4). RDP-classifier tool confirmed their affiliation to the genus *Novosphingobium*. The other two colonies that were originally isolated on plant-only teabags culture media and re-cultivated on Standard I nutrient agar were identified as follows: the isolate LP3.11 affiliated to phyla: Proteobacteria, class: Gammaproteobacteria, closely matching to an uncultured Bacterium (HF586982.1) and to *Lysobacter pocheonensis* (EU273938.1); the isolate LP2.2 affiliated to phyla: Bacteriodetes, class: Sphingobacteria, closely matching to the uncultured *Pedobacter* sp. (JN697525.1) (Fig 4). These two colonies most likely represent a fraction of fastidious rhizobacteria that were not originally developed on Standard I nutrient agar, being possibly overgrown by a faster growing population. In contrast, the plant-based culture media provided them with an appropriate nutrient matrix (in terms of complexity, concentration and diversity) which allowed such a distinct fraction to be unmasked upon culturing.

## Discussion

Throughout the recent history of microbiological research, different types of culture media have been designed to meet the nutritional requirements of microorganisms cultured *in vitro*. While these media have been mostly successful for the general cultivation of a broad range of microorganisms, they have been less successful when it comes to meeting the requirements for cultivating endophytes. We have addressed this issue by using a novel approach which focuses on the use of plant based culture media for the recovery and cultivation of bacterial endophytes. The proposed plant-only culture medium is of a complex nature that mimics the nutritional matrix present in the plant root and/or rhizosphere. Such plant-only-based culture media contain relatively well-balanced amounts of nutrients [9–11], being of natural origin, concentration and diversity. We found that a culture medium with concentrations as low as 0.25 g plant powder per liter supported the cultivability of a large number of Lucerne rhizobacteria, to the extent that no statistical significant differences were observed between the tested concentrations of plan-only teabags culture medium (0.25, 0.5, 1.0, 4.0 g l^-1^). The population densities of rhizobacteria, in terms of total CFUs, were higher on plant-only teabags media compared with the rich Standard I nutrient agar; recovering up to 62–71% and 2–16% of the total bacterial numbers (as measured by qPCR), respectively. This result corresponds with a number of other techniques that have been proposed to develop culture media with low nutrient concentrations in order to facilitate culturing the unculturables [30–32]. Further, the community composition of rhizobacteria recovered on plant-only teabags culture media was substantially different, being clustered apart from those fed on Standard I nutrient agar. In fact, the use of plant-only teabags culture media resulted in the development of more diverse populations of Lucerne rhizobacteria and extended the range of cultivability among its microbiome. This enabled us to easily recover pure isolates of globally distributed rhizobacteria, predominantly *Sinorhizobium meliloti*. It appeared that the plant-only teabags culture media gave preference to culturability and recovery of the specific micro-symbiont of Lucerne, namely *S*. *meliloti*. Moreover, they were able to reveal fastidious not-yet-cultured communities of Lucerne rhizobacteria (Table 2). Both of our two isolates LP1.2 and LP3.2 represent a high similarity to the uncultured Bacterium (AM697055) detected in the dust of indoor environments in Finland [33], and the uncultured *Novosphingobium* sp. (HM438566) which have been detected in a PCR-amplified 16S rDNA sequence from Mexican soil [34]. Two more isolates were originally isolated on plant-only teabags culture media and were subsequently able to regenerate on Standard I nutrient agar. The isolate LP3.11 was most closely (99% similarity) related to an uncultured bacterium (HF586982.1), detected in a batch sample of activated sludge (Germany), and *Lysobacter pocheonensis* (accession no. EU273938) isolated from the soil of ginger and ginseng fields in South Korea and China. The second isolate LP2.2 was found to be most closely related (99% similarity) to an uncultured *Pedobacter*- JN697525 [35], and the nearest cultivated type strain is *Pedobacter steynii* isolated from a spring of the Westerhofer Bach, Westerhof, Germany [36]. So far and to the best of our knowledge, those 3 genera (*Novosphingobium*, *Lysobacter*, and *Pedobacter*) brought into cultivation via plant-only teabags culture media were not reported in literature as culturable endophytes of Lucerne.

**Table 2:** Growth performance of a group of rhizobacteria isolates of Lucerne, originally developed on Lucerne-powder culture medium, on commonly-used chemically-synthetic culture media.

Isolates (codes)	Culture media of original isolation	Successive growth on other culture media	Comments
**Rhizobia isolates**			
***S*. *meliloti*** - LP1.1, LP1.3, LP3.1, LP3.3, LP3.4, LP3.5, LP3.7, and LP3.8	Plant medium (+)	NA (-)	*In vitro* free-living growth of *S*. *meliloti* requires growth factors (amino acids/vitamins [37]) that are naturally present with balanced amounts in the plant medium, compared to ambiguous quantities in the yeast extract added to the chemically-synthetic culture media of YEMA, LB, TY [37]
		CCM (-)	
		YEMA (+/-)	
Non-rhizobia isolates*			
***Novosphingobium* sp**. - LP1.2 and LP3.2	Plant medium (+)	NA (-)	As root stimulating bacteria, cultivable isolates of *Novosphingobium* sp. brought to culture in wood plant culture medium (WPM) amended with casein hydrolysate, nicotinic acid, pyridoxine, thiamine, glycine, asparagine and glutamine [38]
		CCM (-)	
		YEMA (+/-)	
***Pedobacter* sp**. - LP2. 2	Plant medium (+)	NA (+)	To optimize growth of culturable isolates of *Pedobacter* sp., rich amendments of tryptone, yeast extract, and NH_4_Cl are required [39]
		CCM (-)	
		YEMA (+)	
***Lysobacter* sp**. - LP3.11	Plant medium (+)	NA (+)	To bring the very low populations of terrestrial and aquatic *Lysobacter* sp. into cultivation, need to be enriched in culture media containing yeast cells, in addition to antibacterial and antifungal drugs inhibiting other microorganisms [40]
		CCM (-)	
		YEMA (+)	

+, good growth; +/-, scant growth; -, no growth. NA, nutrient agar. YEMA, yeast extract mannitol agar, representing a number of yeast extract-containing culture media (LB, TY, R2A) commonly used to culture rhizobia. CCM, N-deficient combined carbon sources culture medium used for culturing rhizobacteria other than rhizobia, grouped with similar culture media, e.g. M9, and supplemented with yeast extract and/or defined amino acids/vitamins.

*Such non-rhizobia three genera, representing many other fastidious rhizobacteria associated/satellite to rhizobia, emerged/enriched on the plant culture media because of its contents of a wide array of natural amounts of C and N compounds as well as growth factors (amino acids and vitamins); But, they are smeared/masked by rather big and slimy colonies of fast-growing bacteria invasive to chemically-synthetic culture media (NA and YEMA,LB, TY, TSA, R2A) because of their copious contents of peptones, casein, beef and yeast extracts, sugars, alcohols,…etc.

It is known that free-living growth of *S*. *meliloti* is somewhat problematic, as they reported to require growth factors, such as amino acids, i.e. methionine, cysteine, and histidine as well as members of vitamin B group [37]. These nutritional demands are usually met via the inclusion of yeast extract in the commonly used rich chemically-synthetic culture media, e.g. YEMA, LB, and TY. The major problem of using such rich yeast extract-amended culture media is the unusual over growth of big slimy colonies of rhizobia as well as other fast-growing bacteria, mainly representatives of Firmicutes and Gammaproteobacteria that smear or mask the satellite fastidious rhizobacteria [38–40]. The latter group of rhizobacteria may feebly develop in culture media of lower concentrations of nutrients, e.g. N-deficient combined carbon sources medium (CCM, [41]), as they still require essential growth factors. This is not the case when using the plant-based culture medium because of the balanced Lucerne’s biochemical repertoire, having proteins (>3.0–5.0%), Carbohydrates (>6.0–17.0%), 7 essential and 11 non-essential amino acids (>0.05–0.56%); in addition to macro- (>0.3–6.0%) and micro- (>13.0–70.0 μg/g) nutrients [42]. This particular nutritional matrix mimics the root milieu, and exceptionally satisfies both growth requirements, not only of specific rhizobia but also of other associated rhizobacteria, especially those hard to culture, e.g. the species of *Lysobacter* sp., *Pedobacter* sp., and *Novosphingobium* sp. that we reported for first time among the culturable microbiome of Lucerne.

We further used the G3 PhyloChip microarray to characterize the composite culturable rhizobacteria populations developed on culture media (unpublished data). Compared to nutrient agar, populations recovered on plant-based culture media included several unculturable microorganisms of the phyla Acidobacteria, Actinobacteria, Armatimonadetes, Chlorobi, Chloroflexi, Cyanobacteria, Gemmatimonadetes, Planctomycetes, Tenericutes, Synergistetes, Fibrobacteres, and Fusobacteria. We even found a greater culturability and abundance of representatives of the previously unculturable candidate phyla Atribacteria (OP9), Dependentiae (TM6), Gracilibacteria (GN02), Latescibacteria (WS3) Omnitrophica (OP3) and BRC1.

In conclusion, the data presented here, together with those reported earlier [9–11], strongly support the use of plant-only-based culture media as a new method for culturing rhizobacteria. It opens a new horizon towards exploring the unculturable world of authentic plant-fed rhizobacteria, with the possibility of uncovering bacteria with a great potential for improving plant nutrition and health. We are following in the footsteps of one of the great microbiology pioneers Hans Christian Gram (1853–1938), who stated that “the method is published, although we are aware that as yet it is very defective and imperfect; but it is hoped that in the hands of other investigators it will turn out to be useful” (schaechter.asmblog.org/).
